# Machine learning methods for adult OSAHS risk prediction

**DOI:** 10.1186/s12913-024-11081-1

**Published:** 2024-06-05

**Authors:** Shanshan Ge, Kainan Wu, Shuhui Li, Ruiling Li, Caizheng Yang

**Affiliations:** 1 Health Management Center, the First Hospital of Shanxi Medical University, Taiyuan, 030001 China; 2 Nursing College of Shanxi Medical University, Taiyuan, 030001 China

**Keywords:** Machine learning, Obstructive sleep apnea hypopnea syndrome, Prediction

## Abstract

**Background:**

Obstructive sleep apnea hypopnea syndrome (OSAHS) is a common disease that can cause multiple organ damage in the whole body. Our aim was to use machine learning (ML) to build an independent polysomnography (PSG) model to analyze risk factors and predict OSAHS.

**Materials and methods:**

Clinical data of 2064 snoring patients who underwent physical examination in the Health Management Center of the First Affiliated Hospital of Shanxi Medical University from July 2018 to July 2023 were retrospectively collected, involving 24 characteristic variables. Then they were randomly divided into training group and verification group according to the ratio of 7:3. By analyzing the importance of these features, it was concluded that LDL-C, Cr, common carotid artery plaque, A1c and BMI made major contributions to OSAHS. Moreover, five kinds of machine learning algorithm models such as logistic regression, support vector machine, Boosting, Random Forest and MLP were further established, and cross validation was used to adjust the model hyperparameters to determine the final prediction model. We compared the accuracy, Precision, Recall rate, F1-score and AUC indexes of the model, and finally obtained that MLP was the optimal model with an accuracy of 85.80%, Precision of 0.89, Recall of 0.75, F1-score of 0.82, and AUC of 0.938.

**Conclusion:**

We established the risk prediction model of OSAHS using ML method, and proved that the MLP model performed best among the five ML models. This predictive model helps to identify patients with OSAHS and provide early, personalized diagnosis and treatment options.

## Introduction

Obstructive sleep apnea hypopnea syndrome (OSAHS) is an important disease in the current medical environment. It affects about 5% of the population [1]. However, it is estimated that this is a highly undiagnosed disease [2]. The clinical features of the disease are snoring during sleep, repeated episodes of upper respiratory tract failure resulting in intermittent hypoxia, accompanied by symptoms of sleep fragmentation and daytime sleepiness [3]. It is associated with chronic cardiovascular disease, type 2 diabetes, kidney damage, etc. [4–6]. The physiological and pathologic mechanisms of OSAHS are not well understood, but may be multifactorial, including sympathetic nervous system overactivity [7], selective activation of inflammatory pathways [8], oxidative stress [9], vascular endothelial dysfunction [10], and metabolic disorders, particularly involving insulin resistance [11] and lipid metabolism disorders [12], which may further increase cardiovascular risk [13]. Epidemiological studies have shown that OSAHS is independently associated with alterations in glucose metabolism and puts patients at increased risk of developing type 2 diabetes [14, 15]. Although several studies have reported an association of OSAHS with hyperleptinemia, the adjustment of obesity and visceral fat distribution has been inconsistent [16]. In addition, it has been reported that body mass index (BMI) is an important confounder of the relationship between OSAHS and leptin levels [17].

Nocturnal polysomnography (PSG) is one of the standard tests for diagnosing OSAHs [18]. However, PSG has certain limitations: 1.PSG requires professional medical personnel to operate, and patients must be monitored in the hospital; 2. Patients need to install corresponding devices in many parts of the body, which affects the quality of sleep [19]. If there is a method that does not rely on PSG to assess whether snoring patients have OSAHS, it will be easier to help diagnose OSAHS.

The fields of artificial intelligence (AI) and machine learning (ML), are moving in this direction. In recent years, machine learning has been widely developed and applied in the medical field with its excellent performance [20–22]. It can extract information from complex non-linear data and build models that reveal hidden dependencies between factors and disease in a big data environment [23]. According to different symptom characteristics, scholars have proposed a variety of OSAHS disease identification techniques. For example, Bruno Arsenali et al. develop an algorithm for classification of snoring and non-snoring sound events [24]. Takahiro Emoto classified snoring data related to OSAHS (SNR) based on MLP [25].

Considering OSAHS is associated with a variety of chronic diseases, and these inextricable associations will be reflected in all aspects of the human body, we attempt to identify OSAHS patients with the simplest health management data. We apply ML methods to the diagnosis of OSAHS, such as: logistic regression, support vector machines, Boosting, Random Forest, MLP, etc. This work, which has never been done before, provides a valuable tool for clinicians to assess the risk of OSAHS.

## Materials and methods

### Study design and subjects

The study retrospectively analyzed 2,166 patients with snoring who underwent physical examinations at the Health Management Center of the First Affiliated Hospital of Shanxi Medical University from July 2018 to July 2023, of whom 102 patients had partial data missing and were discarded. All of the remaining study subjects underwent PSG monitoring, blood testing and carotid artery ultrasound. The AHI (Apnea Hypopnea Index) value of the patient was obtained by monitoring the patient's breathing overnight through PSG, so that the patient's symptoms could be graded. OSAHS was diagnosed with AHI ≥ 5 times/hour in PSG results [26]. Patient details can be found in the supplement materials.

The inclusion criteria were as follows: patients with age ≥ 18 years; snoring during night sleep; the patient had received PSG monitoring, blood tests and carotid ultrasound; patients who have not received OSAHS-related treatment; patients sign informed consent forms. The exclusion criteria were as follows: patients with incomplete baseline data; the patient had no snoring symptoms; patients with multiple organ dysfunction syndrome, uremia, severe cardiac heart failure, renal, or cardiac transplantation; pregnant women; the patients did not sign the consent form.

### Dataset description

Twenty four relevant clinical indicators were collected, and a total of 24 candidate variables were included as follows:

- Age (years): This feature refers to the age of a person who is over 18 years old. It is numerical data.
- Gender: This feature refers to a person's gender. The number of men is 1628 (78.88%), while the number of women is 436 (21.12%). It is nominal data.
- Height (cm): This feature refers to the height of a person. It is nominal data.
- Weight (kg): This feature refers to the weight of a person. It is nominal data.
- BMI (kg/m^2^): This feature is calculated as weight/height^2^. It is numerical data.
- RBC (10^12^/L): This feature refers to the count of red blood cells in the blood. It is numerical data.
- PLT (10^9^/L): This feature refers to the count of platelets in the blood. It is numerical data.
- MPV (fL): This feature refers to the mean volume size of platelets in the blood. It is numerical data.
- PDW (fL): This feature refers to the dispersion of platelet volume size in the blood. It is numerical data.
- HB (g/L): This feature refers to the mass of hemoglobin per unit volume of blood. It is numerical data.
- HCT (%): This feature refers to the volume of red blood cells per unit volume of blood. It is numerical data.
- TC (mg/dL): This feature captures the participant's total cholesterol. It is numerical data.
- TG (mg/dL): This characteristic refers to the mass of triglycerides per unit volume of blood. It is numerical data.
- LDL-C (mmol/L): This feature captures the participant's low-density lipoprotein. It is numerical data.
- HDL-C (mmol/L): This feature captures the participant's high-density lipoprotein. It is numerical data.
- A1c (%): This feature refers to the proportion of glycosylated hemoglobin. It is numerical data.
- SBP (mmHg): This feature captures the participant’s systolic blood pressure. It is numerical data.
- DBP (mmHg): This feature captures the participant’s diastolic blood pressure. It is numerical data.
- GPT (U/L): This feature refers to the patient's alanine aminotransferase. It is numerical data.
- TBIL (μmol/L): This feature refers to the patient's total bilirubin. It is numerical data.
- GGT(U/L): This feature refers to the patient's glutamyltranspeptidase. It is numerical data.
- Cr (μmol/L): This feature refers to the patient's creatinine. It is numerical data.
- UA (μmol/L): This characteristic refers to the patient's uric acid. It is numerical data.
- Common carotid artery plaque: This feature refers to the presence or absence of carotid artery plaque in the patient. It is categorical data.

### Features ranking

Screening features to further reduce the number of features used to generate predictive models can help mitigate the risk of model overfitting [27]. Among several feature selection methods, we focus on the feature-based ranking method, which ranks each feature in descending order of importance, and we consider the following methods. First, we apply the Pearson correlation coefficient [28] to assess the strength of association between all features. We then apply the Information Gain method (InfoGain), which assesses the value of a feature by measuring the information gain relative to the class. And we employed the Gain Ratio (GR) method, which indicates the relevance of a feature and selects the ones that maximize gain ratio based on the probability of each feature value [29]. In addition, we use the built-in random forest feature importance function in sklearn library and use the sns.barplot() function to sort and output features according to their importance.

### Machine learning models

**Fig. 1:**
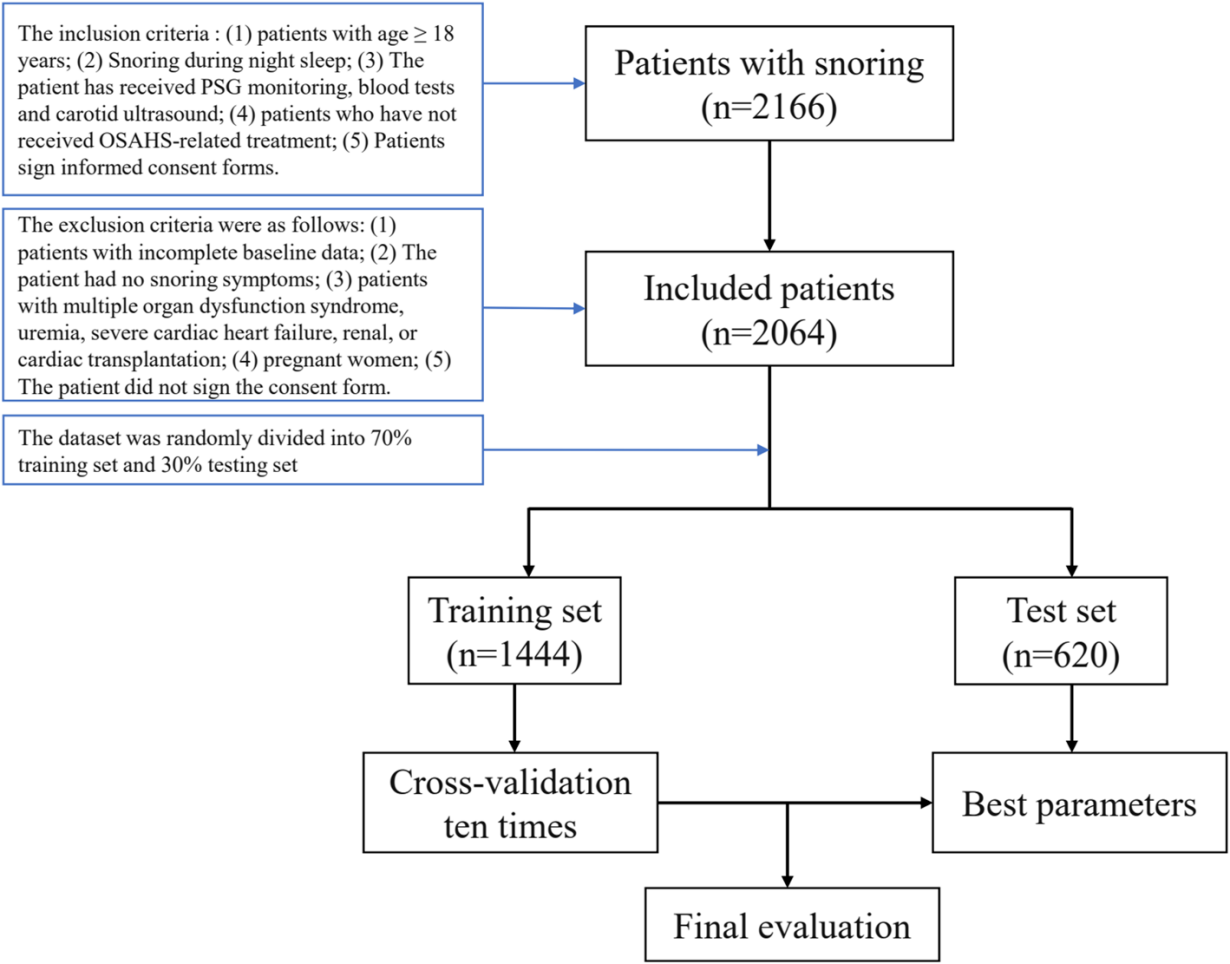
Overview of our machine learning process

All patients were randomly divided in a 7:3 ratio into a training set for building the predictive model and a test set for model validation. Five representative supervised machine learning algorithms were selected for model construction in the training dataset: support vector machine, random forest, boosting, logistic regression, artificial neural network. The working characteristics of the subjects of five ML models were plotted using the test set, and cross validation was used to adjust the model hyperparameters. The AUC values were calculated to evaluate the predictive ability of different models in the cohort. By comparing the predictive performance of machine learning models, the model with the best predictive performance is selected as the final model. In addition, confusion matrix was used to evaluate the performance of the prediction model. The specific process is shown in Fig. 1.

#### Logistic regression

Logistic Regression (LR) [30] logistic regression is intended for binary (two-class) classification problems. It will predict the probability of an instance belonging to the default class, which can be snapped into a 0 or 1 classification.

#### Random forest

Random Forest (RF) [31] is a combination of classification trees determined by randomly selected samples and has good robustness. However, their performance is strictly related to a number of parameters, including the number of trees in the forest and pruning strategies.

#### Support vector machine

Support Vector Machine (SVM) [32] use a set of mathematical functions that are defined as the kernel. The function of kernel is to take data as input and transform it into the required form. Different SVM algorithms use different types of kernel functions. These functions can be different types. For example, linear, nonlinear, polynomial, radial basis function (RBF), and sigmoid. SVM perform classification by finding linear decision boundaries that are as far away from the data as possible. They work great with linearly separable data.

#### Boosting

Boosting [33] is a very powerful learning method, and it's also a supervised categorical learning method. It combines many "weak" classifiers to produce a strong group of classifiers. A weak classifier performs only slightly better than random selection, so it can be designed to be very simple and not too computationally expensive. Many weak classifiers are combined to form an integrated strong classifier similar to SVM or neural network. Three of the most famous algorithms include AdaBoost, Gradient Boosting Machine, and XGBoost.

#### Multilayer perceptron model

A Multilayer Perceptron model (MLP) [34] is a standard fully connected neural network model. It consists of input and output layers and at least one hidden layer. Its neurons are trained by employing back-propagation learning which allows for classification into multiple labels.

### Evaluation metrics

To evaluate the performance of ML models, we measured accuracy, precision, recall, F1-score, and AUC metrics [35]. Confusion Matrix can help us better understand and calculate the above metrics. The confusion matrix is shown below:

**Table:** 

*Confusion*		*Predicted value*	
		*Positive*	*Negative*
*True value*	*Positive*	*TP (True Positive)*	*FN (False Negative)*
	*Negative*	*FP (False Positive)*	*TN (True Negative)*

TP (True Positive): indicates the positive example that is correctly predicted. That is, the real value of the data is a positive example, and the predicted value is also a positive example

TN (True Negative): A negative example of being correctly predicted. That is, the real value of the data is a negative example, and the predicted value is also a negative example

FP (False Positive): indicates the positive example that is incorrectly predicted. That is, the real value of the data is a negative example, but it is wrongly predicted as a positive example

FN (False Negative): A negative example of being incorrectly predicted. That is, the true value of the data is a positive example, but it is incorrectly predicted to be a negative example

Accuracy represents the proportion of correctly classified samples in the total number of samples.

$$Accuracy\hspace{0.17em}=\hspace{0.17em}(TN\hspace{0.17em}+\hspace{0.17em}TP)/(TN\hspace{0.17em}+\hspace{0.17em}TP\hspace{0.17em}+\hspace{0.17em}FN\hspace{0.17em}+\hspace{0.17em}FP)$$

Precision Indicates the percentage of the predicted positive samples that are actually positive.

$$Precision\hspace{0.17em}=\hspace{0.17em}TP/(TP\hspace{0.17em}+\hspace{0.17em}FP)$$

Recall indicates the proportion of the actual number of positive samples in the total positive samples in which the prediction result is positive.

$$Recall\hspace{0.17em}=\hspace{0.17em}TP/(TP\hspace{0.17em}+\hspace{0.17em}FN)$$

The F1-score is a weighted average of accuracy and recall rates.

$$F1\hspace{0.17em}=\hspace{0.17em}2*Precision*Recall / (Precision\hspace{0.17em}+\hspace{0.17em}Recall)$$

Support is the number of samples for each category or the total number of samples for the test set.

An ROC curve (receiver operating characteristic curve) is a graph showing the performance of a classification model at all classification thresholds. This curve plots two parameters: True Positive Rate and False Positive Rate. AUC stands for "Area under the ROC Curve." That is, AUC measures the entire two-dimensional area underneath the entire ROC curve (think integral calculus) from (0,0) to (1,1).

### Statistical analysis

All establishment and analyses of models were performed with Python software (version 3.7.6). Statistical analysis of the clinical data was performed using SPSS for Windows (version 23). For the demographic and clinical evaluation data, SPSS 23.0 software was first used for processing, and kolmogorov–Smirnov was used to test the normality of the data. Then, Student's t-test was performed on the data conforming to normal distribution, and Mann–Whitney U test was performed on the non-normal distribution data. Chi-square test was adopted for the data of dichotomous variables. *P* < 0.05 was considered statistically significant.

## Results

### Demographic and clinical characteristics

The demographic and clinical characteristics of OSAHS group and non-OSAHS group are summarized in Table 1. We found significant differences in gender, BMI, PDW, A1c, SBP, DBP and Cr between OSAHS group and non-OSAHS group (*P* < 0.05).

**Table 1:**
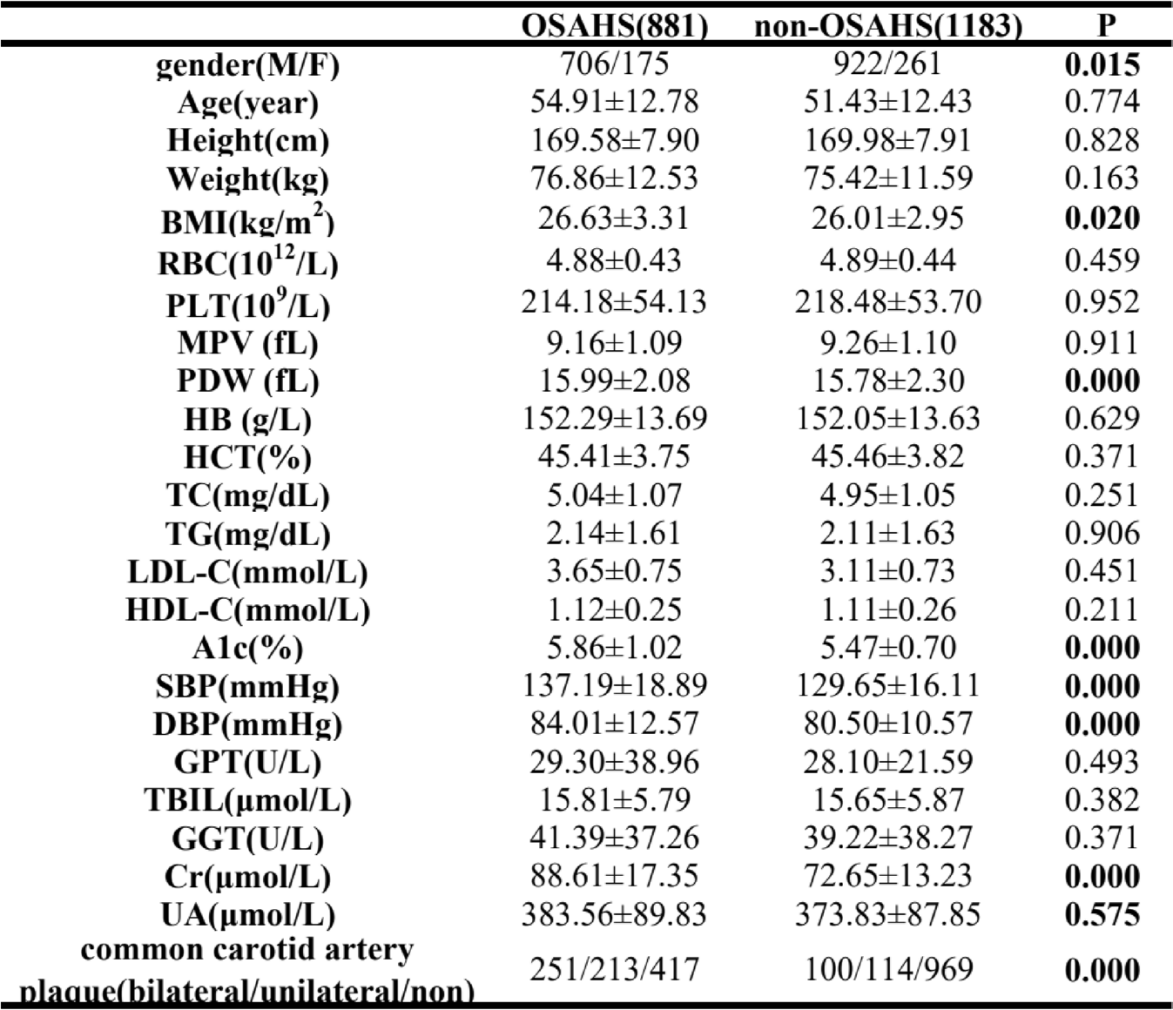
Demographic characteristics of snoring patients in OSAHS and non-OSAHS retrospective datasets for machine learning model training

The differences between OSAHS group and non-OSAHS group were compared for 24 features, using Student's t test, and the data were expressed as mean ± SD. The results showed significant differences in gender, BMI, PDW, A1c, blood pressure, Cr, UA, and common carotid artery plaque between the two groups

*BMI* Body Mass Index, *RBC* Red Blood Cell, *PLT* Platelet, *MPV* Mean Platelet Volume, *PDW* Platelet Distribution Width, *HB* Hemoglobin, *HCT* Hematocrit, *TC* Total Cholesterol, *TG* Triglycerides, *LDL-C* Low-Density Lipoprotein Cholesterol, *HDL-C* High density lipoprotein cholesterol, *A1c* HbA1-glycosylated hemoglobin, *SBP* Systolic Blood Pressure, *DBP* Diastolic Blood Pressure, *GPT* Glutamic-pyruvic Transaminase, *TBIL* Total Bilirubin, *GGT* Gamma Glutamyl Transpeptidase, *Cr* Creatinine, *UA* Uric Acid

### Feature ranking

**Fig. 2:**
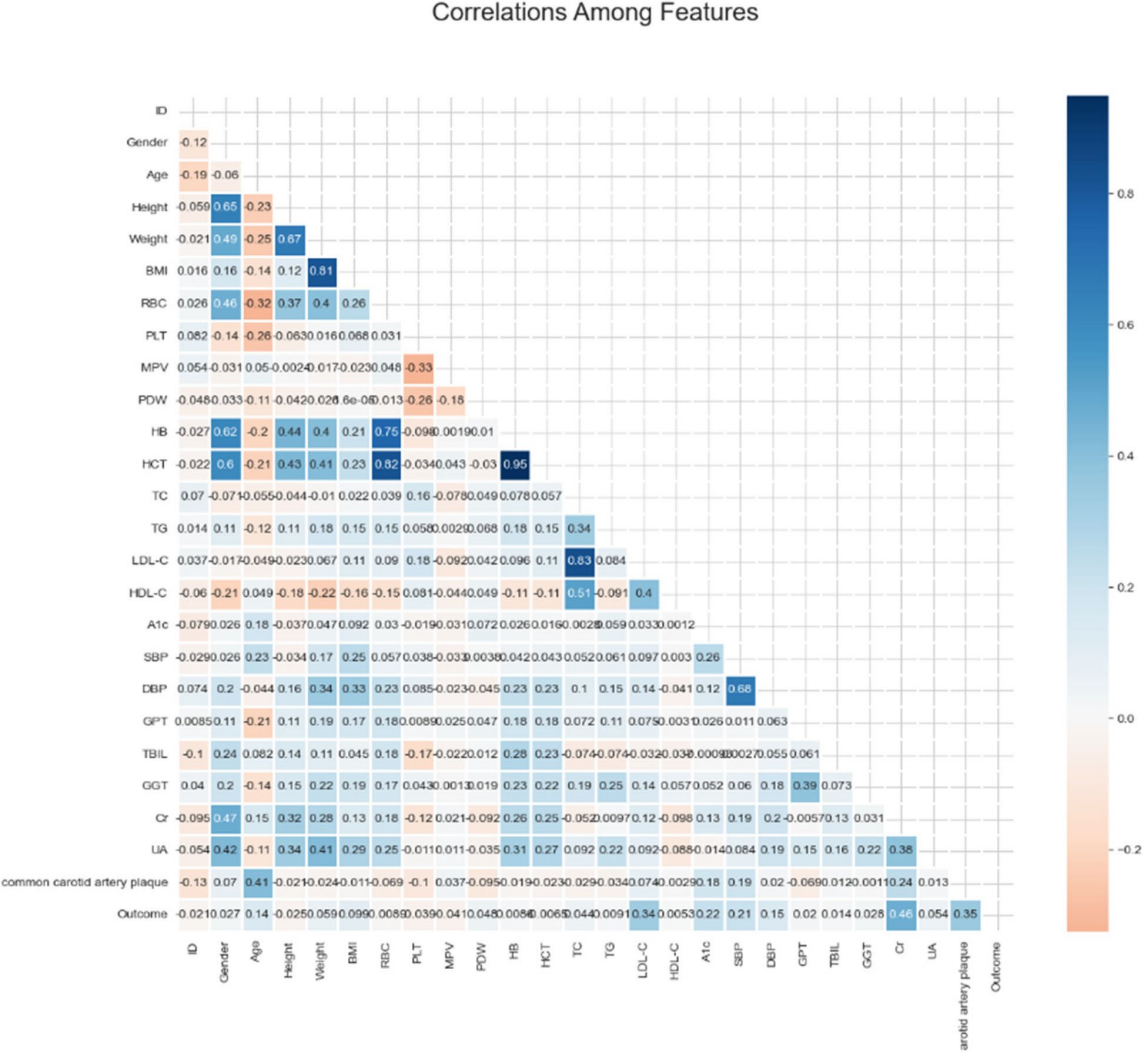
Correlation heat map between 24 features

**Fig. 3:**
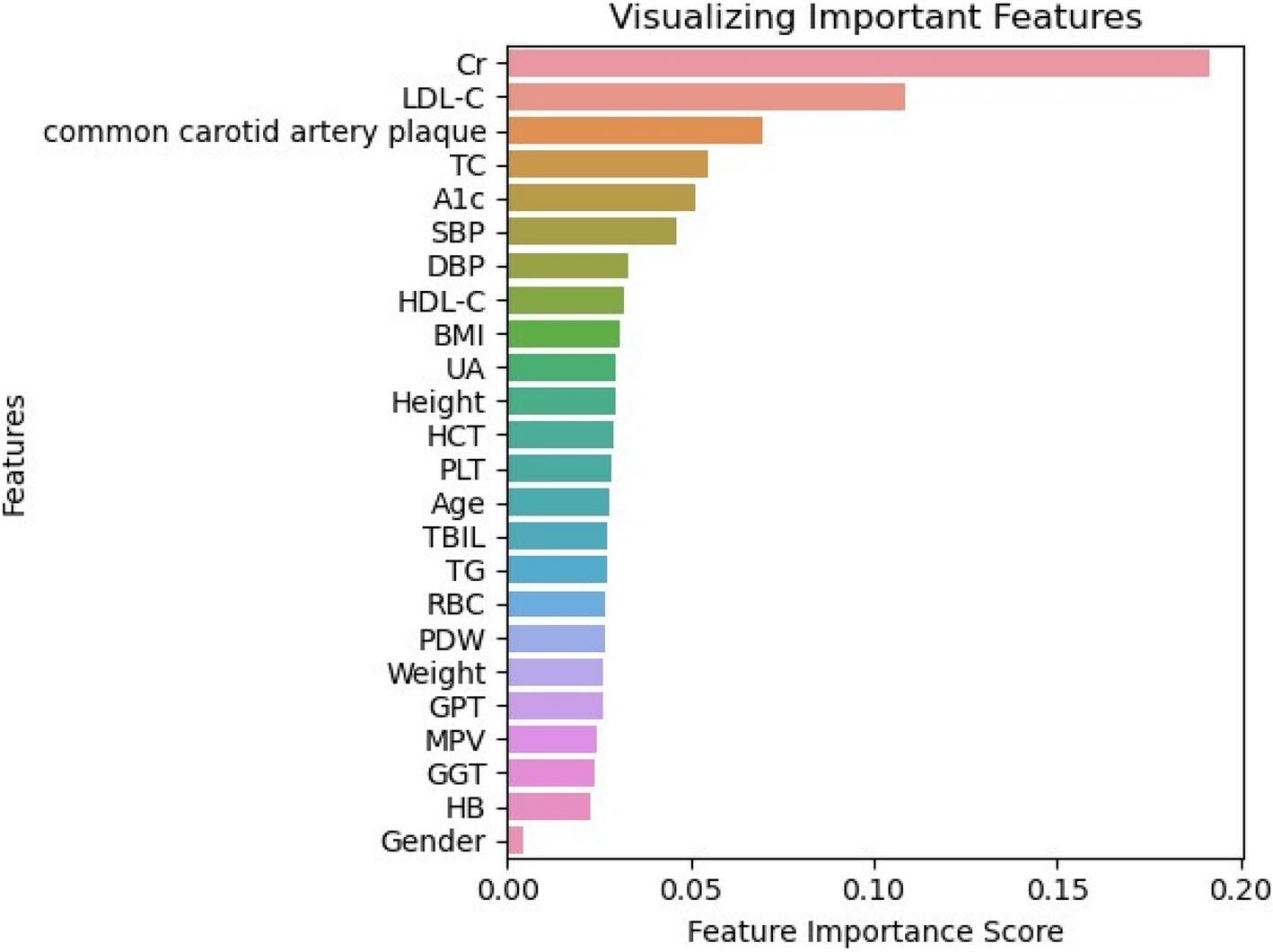
Importance ranking of 24 features

The heat map in Fig. 2 shows the results of the correlation analysis. In the correlation matrix, we observed that the linear relationship between HCT and HB was 0.95, the linear relationship between LDL-C and TC was 0.83, the linear relationship between HCT and RBC was 0.82, the correlation between BMI and Weight was 0.81, the linear relationship between RBC and HB was 0.75. Then, we ranked the importance of features. In Fig. 3 and Table 2, we summarized the importance of features in the balanced data set about OSAHS. All methods considered indicated that LDL-C, Cr, carotid plaque, A1c, and BMI characteristics were more important in the differential diagnosis of OSAHS.

**Table 2:**
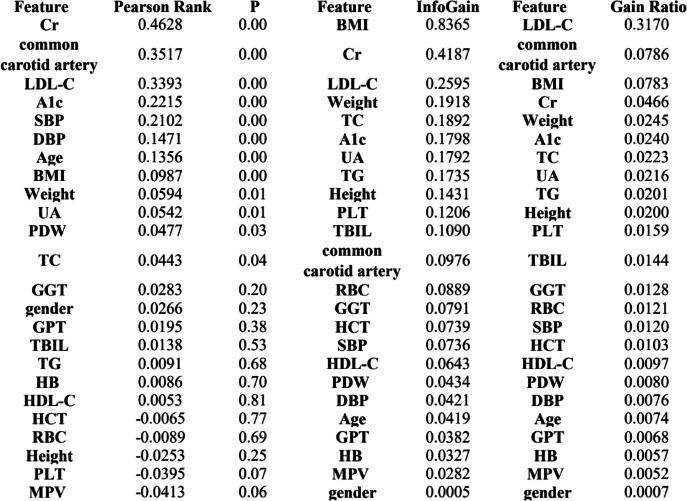
Features’ order of importance

### Evaluation

**Fig. 4:**
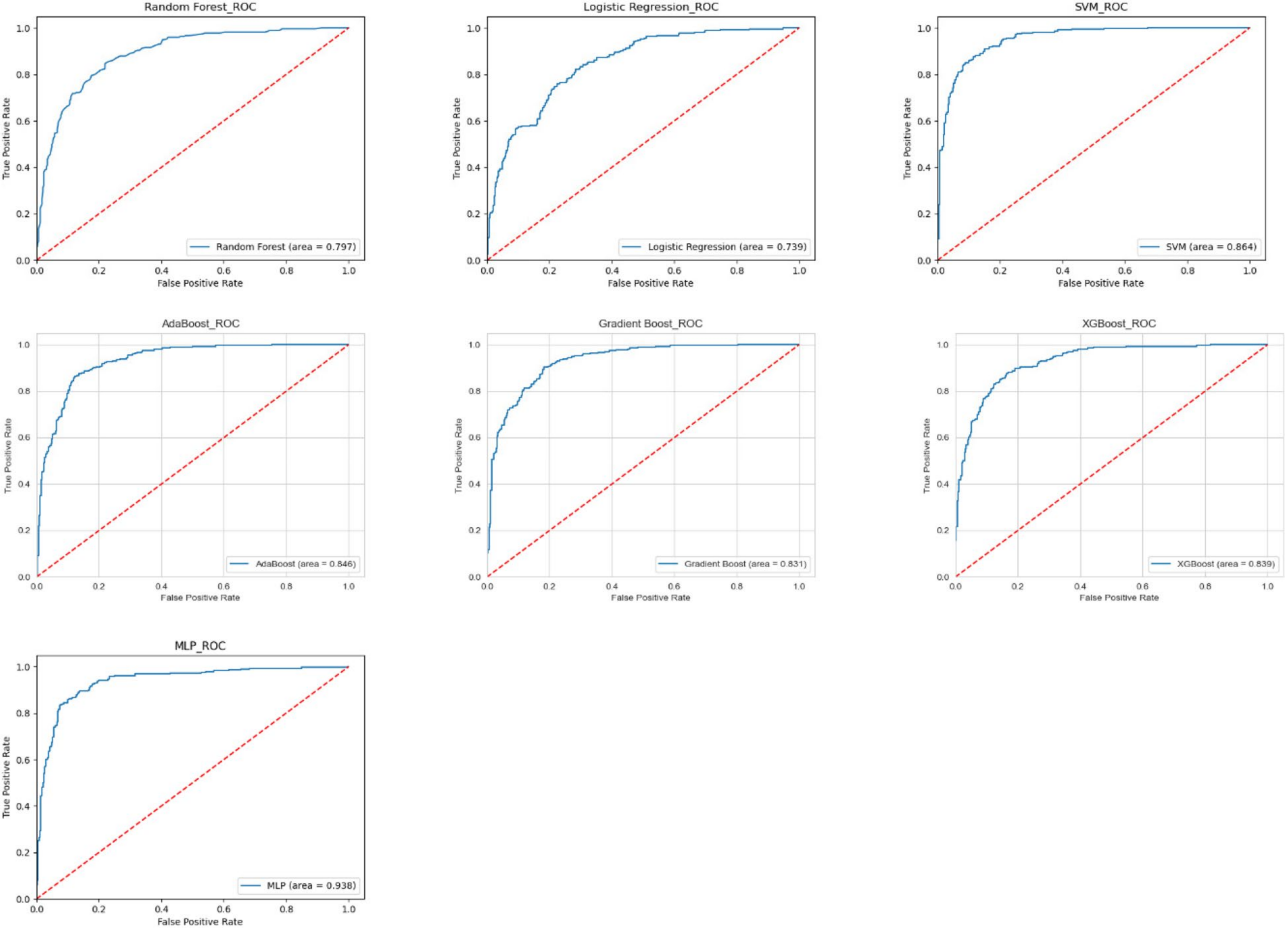
ROC curve of the models

In order to fully evaluate the effectiveness of the model, we use accuracy, precision, recall rate, F1-score and other indicators to evaluate the model. Among all models, Logistic Regression’s accuracy is 75.00%, precision is 0.73, recall is 0.66, F1-score is 0.69, and AUC is 0.739. Support Vector Machine’s accuracy is 87.10%, precision is 0.89, recall is 0.81, F1-score is 0.85, and AUC is 0.864. Gradient Boosting’s accuracy is 83.90%, precision is 0.85, recall is 0.76, F1-score is 0.81, and AUC is 0.831. Adaboost’s accuracy is 85.32%, precision is 0.86, recall is 0.79, F1-score is 0.83, and AUC is 0.846. Xgboost’s accuracy is 84.70%,precision of 0.86, recall of 0.77, F1-score of 0.82, and AUC of 0.839. Random Forests’ accuracy is 80.81%, precision is 0.83, recall is 0.70, F1-score is 0.76, and AUC is 0.797. MLP’s accuracy is 85.80%, precision of 0.89, recall of 0.75, F1-score of 0.82, and AUC is 0.938. Compared these models, SVM and MLP performed well in various evaluation indicators, but the AUC of MLP was higher. MLP performed best across all metrics, so we finally concluded that MLP was the better model. To predictive performance of the best models and the average data after ten different iterations are summarized for each of the five algorithms with five evaluation measures in Table 3. Fig. 4 shows the ROC curves of all models.

**Table 3:**
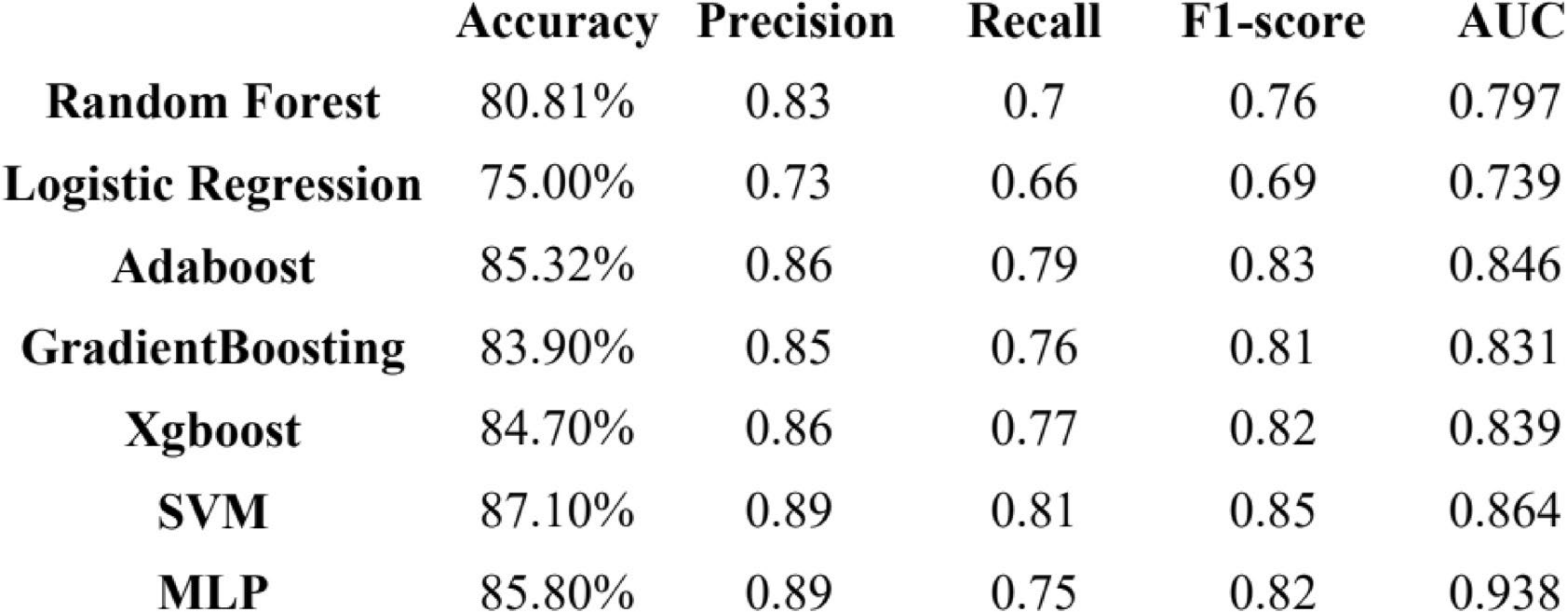
Performance Evaluation of ML Models

## Discussion

In this study, in order to simplify the model and avoid over-fitting, we first conducted a correlation analysis. And then, we assessed the importance of 24 features to OSAHS, and we were surprised to find that LDL-C, Cr, carotid plaque, A1c and BMI made major contributions to OSAHS. At the same time, combined with the feature importance ranking, we find that there was no strong correlation between the top five in the feature ranking. Studies have shown an increased prevalence of OSAHS in patients with metabolomics, chronic kidney disease, carotid plaques, diabetes and obesity [36–40], which is consistent with our findings. Importantly, we evaluated the ability of ML models created by five algorithms to distinguish between OSAHS and non-OSAHS diseases, namely the commonly used Random Forest, boosting, SVM, and logistic regression and MLP methods. Further, we obtained relatively good results for all five models by adjusting the algorithm and selecting the best parameters using GridSearchCV. Among them, the MLP of the five best models appeared to outperform the others, with an accuracy of 85.80%, a Precision of 0.89, a Recall of 0.75, an F1-score of 0.82, and an AUC of 0.938. Although the SVM model also performed well, due to its lower AUC value than the MLP, we ultimately concluded that the MLP performed better. A high AUC score means that the model does a good job of distinguishing between positive and negative samples, and ROC-AUC is a very useful metric when we want to assess the model's ability to distinguish between categories. It is not affected by the selection of thresholds, and can comprehensively consider the performance of the model under various thresholds.

An artificial neural network (ANN) is a computational structure that functions like a biological neuron and can make predictions through computational analysis of massive data sets. There are several types of artificial neural networks, of which MLP is a feedforward network [41]. MLP has the advantage of being easy to implement, providing high-quality models, and having relatively short training times [42]. The MLP network consists of an input layer, one or more hidden layers, and an output layer. Each layer has several processing units (neurons), each of which is weighted to the units in the next layer. A feedforward network with enough neurons in the hidden layer can fit any finite input–output mapping problem. This study successfully constructed MLP network, providing an effective model for snoring patients to further diagnose OSAHS.

There are many reasons for snoring at night, possibly due to metabolic disorders, especially obesity, which is almost 100% involved in snoring; There are also some local anatomic causes and some unverifiable causes [43]. Due to the variety of causes of snoring, and the patients themselves have no subjective cognition of this clinical symptom, the etiology of snoring has not caused enough awareness of patients. Whether these reasons further lead to OSAHS is easy to be ignored. Therefore, our study constructed a simple and feasible model to preliminarily predict the risk of OSAHS.

As a kind of disease that is not easily detected, OSAHS cannot be underestimated [44]. The purpose of this study is to be able to detect the possibility of OSAHS from snoring symptoms combined with health management physical examination, which has not been mentioned in previous studies. At present, the latest research in this field is to collect snoring through non-contact microphone, and establish a high-accuracy model to quantify the snoring events all night. Bruno Arsenali et extracted data from snoring to establish a Recurrent Neural Network model [24], and its’ accuracy was 95%, sensitivity was 92%, and specificity was 98%. Although this model improved the process of snoring detection and obstructive sleep apnea screening, microphones are still needed to monitor the sound emitted during sleep throughout the night, which is still not convenient enough to quickly predict the occurrence of OSAHS. The advantage of our research is that it can help us pay attention to OSAHS earlier and further diagnose OSAHS by specialists, which helps to intervene the disease earlier and get a good prognosis. It is worth noting that our study has achieved good results, we successfully established five LM models, and obtained a more significant performance of MLP, which provides a method guidance for future clinical work. However, this model only aroused doctors' attention and suspicion of OSAHS, and the diagnosis of OSAHS still depended on PSG method. Of course, there are some limitations to this study. Due to the difficulty of data extraction and the absence of data, the sample size and characteristics of this study did not meet the expectations. In our initial framework, we hope to obtain as many examination data as possible, such as common carotid intimedia thickness (CIMT), common carotid blood flow velocity (CBFV), insulin, neck circumference, waist circumference, etc. At the same time, we hope to establish a large OSAHS-related database to train a more optimized model. However, due to different physical examination items or data loss, it is difficult to obtain relevant data for all patients, so the number of patients included in the study is also lower than expected. But our research still produced a satisfactory model, which can use some common symptoms combined with test results to help us predict the presence of OSAHS.

As we all know, machine learning itself is a black box model, and we need to further explain and evaluate the model in future research. In addition, the reproducibility and generalizability of our ML model in OSAHS has not been evaluated because we have not performed external validation.

As mentioned in this paper, this study builds a machine learning model for snoring patients to predict OSAHS on the basis of health management examination, and can help make good predictions, but it cannot completely replace PSG and clinician decision-making, and there is still a chance to become a good assistant to clinicians.

## Data Availability

The datasets analysed during the current study available from the corresponding author on reasonable request.
